# High Potency of Organic and Inorganic Nanoparticles to Treat Cystic Echinococcosis: An Evidence-Based Review

**DOI:** 10.3390/nano10122538

**Published:** 2020-12-17

**Authors:** Aishah E. Albalawi, Abdullah D. Alanazi, Parastoo Baharvand, Maryam Sepahvand, Hossein Mahmoudvand

**Affiliations:** 1 Faculty of Science, University of Tabuk, Tabuk 47912, Saudi Arabia; ae.albalawii@ut.edu.sa; 2 Department of Biological Science, Faculty of Science and Humanities, Shaqra University, P.O. Box 1040, Ad-Dawadimi 11911, Saudi Arabia; aalanazi@su.edu.sa; 3 Department of Social Medicine, School of Medicine, Lorestan University of Medical Sciences, Khorramabad 6813833946, Iran; dr.baharvand@gmail.com; 4 Student Research Committee, Lorestan University of Medical Sciences, Khorramabad 6813833946, Iran; msepahv@gmail.com; 5 Razi Herbal Medicines Research Center, Lorestan University of Medical Sciences, Khorramabad 6813833946, Iran

**Keywords:** hydatid cyst, protoscoleces, nanomedicine, in vitro, in vivo, ex vivo

## Abstract

Since there is no potential, effective vaccine available, treatment is the only controlling option against hydatid cyst or cystic echinococcosis (CE). This study was designed to systematically review the in vitro, in vivo, and ex vivo effects of nanoparticles against hydatid cyst. The study was carried out based on the 06- PRISMA guideline and registered in the CAMARADES-NC3Rs Preclinical Systematic Review and Meta-analysis Facility (SyRF) database. The search was performed in five English databases, including Scopus, PubMed, Web of Science, EMBASE, and Google Scholar without time limitation for publications around the world about the protoscolicdal effects of all the organic and inorganic nanoparticles without date limitation in order to identify all the published articles (in vitro, in vivo, and ex vivo). The searched words and terms were: “nanoparticles”, “hydatid cyst”, “protoscoleces”, “cystic echinococcosis”, “metal nanoparticles”, “organic nanoparticles”, “inorganic nanoparticles, “in vitro”, ex vivo”, “in vivo”. Out of 925 papers, 29 papers including 15 in vitro (51.7%), 6 in vivo (20.7%), ex vivo 2 (6.9%), and 6 in vitro/in vivo (20.7%) up to 2020 met the inclusion criteria for discussion in this systematic review. The results demonstrated the most widely used nanoparticles in the studies were metal nanoparticles such as selenium, silver, gold, zinc, copper, iron nanoparticles (*n =* 8, 28.6%), and metal oxide nanoparticles such as zinc oxide, titanium dioxide, cerium oxide, zirconium dioxide, and silicon dioxide (*n =* 8, 28.6%), followed by polymeric nanoparticles such as chitosan and chitosan-based nanoparticles (*n =* 7, 25.0%). The results of this review showed the high efficacy of a wide range of organic and inorganic NPs against CE, indicating that nanoparticles could be considered as an alternative and complementary resource for CE treatment. The results demonstrated that the most widely used nanoparticles for hydatid cyst treatment were metal nanoparticles and metal oxide nanoparticles, followed by polymeric nanoparticles. We found that the most compatible drugs with nanoparticles were albendazole, followed by praziquantel and flubendazole, indicating a deeper understanding about the synergistic effects of nanoparticles and the present anti-parasitic drugs for treating hydatid cysts. The important point about using these nanoparticles is their toxicity; therefore, cytotoxicity as well as acute and chronic toxicities of these nanoparticles should be considered in particular. As a limitation, in the present study, although most of the studies have been performed in vitro, more studies are needed to confirm the effect of these nanoparticles as well as their exact mechanisms in the hydatid cyst treatment, especially in animal models and clinical settings.

## 1. Background

Hydatid cyst or cystic echinococcosis (CE) is well-known as one of the most common universal parasitic infections, which infects a wide range of hosts such as humans, wild animals, and domestic livestock [1]. Therefore, CE can be considered as an important challenge both from medical and economic points of view [2]. In humans, hydatid cyst occurs through accidental infection with ingesting eggs of *Echinococcus granulosus* (dog tapeworm) expelled from the dog as the final host, followed by the growth of the larvae stage and transformation into cyst, predominantly in the liver (nearly 70%), and less frequently in the lungs, spleen, kidneys, and brain [3]. Considering the clinical symptoms of hydatid cyst, the onset of the disease shows no specific symptoms; but depending on the number, location, and size, the cysts have variable symptoms from mild to deadly [4].

Since there is no potential, effective vaccine available, treatment is the only controlling option against hydatid cyst diseases. Today, the therapeutic approaches for hydatid cyst treatment are medical treatment, surgical treatment, endoscopic interventional treatment, percutaneous methods (puncture, aspiration, injection, and re-aspiration (PAIR)), as well as the consequent minimally invasive techniques [5]. Therefore, in small and inactive cysts, the preferred treatment is chemotherapy with benzimidazole derivatives (mebendazole and albendazole); however, the first choice treatment for large and active cysts is surgery [6].

The results of recent studies have shown chemotherapy with benzimidazole derivatives is associated with some side-effects, i.e., hepatotoxicity, teratogenicity, methemoglobinemia, severe leucopenia, thrombocytopenia, and osteoporosis, indicating that caution should be exercised in using of these drugs [6]. By surgical treatment, since the rupture of cysts or leakage of their contents (protoscoleces) may cause re-infection, secondary infection, as well as anaphylaxis shock, surgeons use a number of chemical protoscolicidal agents such as hypertonic saline 20%, silver nitrate, and formalin to prevent these complications [4]. Hence, recent studies have demonstrated that the current protoscolicidal agents are not risk-free and can cause complications such as biliary fibrosis, hepatic necrosis, and cirrhosis [4,7]. Therefore, searching and discovering a new protoscolicidal agent are of top priority for physicians in this field.

Nanomedicine is considered as a relatively new field of science and technology that deals with nanometer-sized materials for medical purposes [8]. To date, nanomedicine has a variety of diagnostic and therapeutic applications in modern medicine, such as drug delivery, imaging, diagnosis, medical devices, vaccines, as well as antimicrobial therapy [9]. Considering applications of nanomedicine in treating microbial diseases, a wide range of studies have reported the antimicrobial effects of some inorganic nanoparticles (such as metal and metal oxide) and organic nanoparticles (peptide- and polymer-based nanoparticles such as cationic peptides, synthetic cationic polymers, chitosan, etc.) [10,11,12]. Considering the protoscolicidal activity of nanoparticles, although Shnawa et al. [13] reviewed the application of nanomedicine, especially green biosynthesis nanoparticles such as biogenic selenium, silver, gold, and chitosan nanoparticles, as new protoscolicidal alternative to treat hydatid cysts [13], in this study, we aim to systematically review the in vitro, in vivo, and ex vivo effects of a wide range of nanoparticles such as metal, carbon-based nanoparticles, lipid-based nanoparticles, polymeric nanoparticles, etc. against hydatid cyst.

## 2. Materials and Methods

### 2.1. Search Strategy

**Figure 1:**
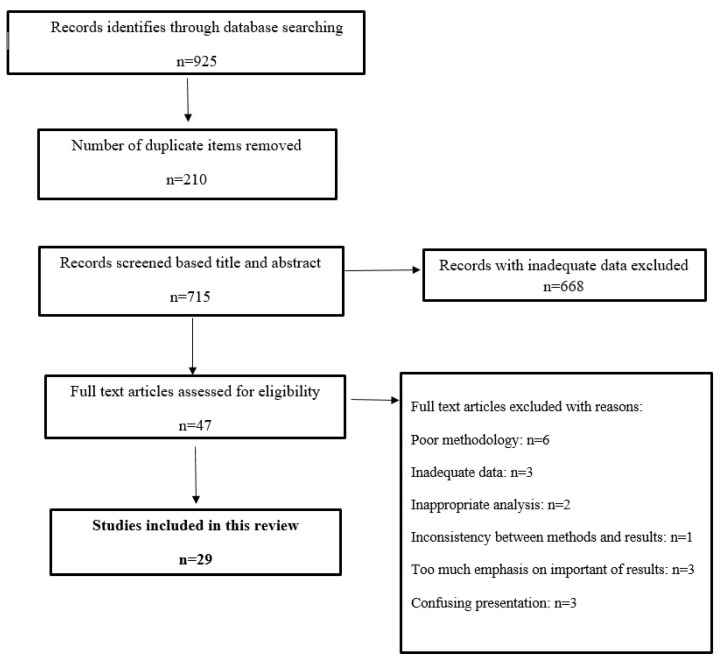
Flowchart describing the study design process.

The current study was carried out based on 06- PRISMA guideline [14] and registered in the CAMARADES-NC3Rs Preclinical Systematic Review and Meta-analysis Facility (SyRF) database. The search was performed in five English databases, including Scopus, PubMed, Web of Science, EMBASE, and Google Scholar without time limitation for publications worldwide on the protoscolicidal effects of organic and inorganic nanoparticles without date limitation in order to identify all the published articles (in vitro, in vivo, and ex vivo). Studies in any languages were entered in the search step if they had an English abstract. The words and terms were used as a syntax with specific tags of each database. The searched words and terms were: “protoscolicidal”, “scolicidal”, “nanoparticles”, “hydatid cyst”, “metal nanoparticles”, “protoscoleces”, “cystic echinococcosis”, “in vitro”, ex vivo”, “in vivo”, “scolex” etc. (Figure 1).

### 2.2. Quality Assessment and Article Selection

Those studies were examined, in which the effects of nanoparticles against hydatid cyst were investigated. First, the studies were imported into EndNote X9 software (Thomson Reuters, New York, NY, USA) and duplicate studies were deleted. Afterwards, three independent authors examined the title and abstract of the studies and relevant works were included for further analysis. The same authors carefully read the studies and the eligible studies with adequate inclusion criteria were selected.

### 2.3. Exclusion Criteria

The studies with inadequate information, abstract submitted in congresses, full texts of which were not available, and failure to match methods with the incorrect interpretation of the results was excluded from the current study.

### 2.4. Inclusion Criteria

Inclusion criteria of this study were the articles evaluating the in vitro, ex vivo, and in vivo effects of various forms of nanoparticles containing drugs and other pharmaceutical formulations of organic and non-organic nanoparticles against hydatid cyst (Figure 1).

### 2.5. Data Extraction

Three independent authors extracted information from the selected articles and, if needed, the differences were resolved by the corresponding author. The extracted data included nanoparticle type, in combination or loaded with other drugs, type of study, animal model, concentration, time of use, reference, etc.

## 3. Results

Out of 925 papers, 29 papers including 15 in vitro (51.7%), 6 in vivo (20.7%), ex vivo 2 (6.9%), and 6 in vitro/in vivo (20.7%) up to 2020 met the inclusion criteria for discussion in this systematic review, the extracted data of which are presented in Table 1, Table 2 and Table 3. The most common type of nanoparticles were organic nanoparticles (15 studies, 51.7%) such as polymeric, lipid, etc., followed by non-organic nanoparticles such as metal and metal oxide nanoparticles (14 studies, 48.3%). The results demonstrated that the most widely used nanoparticles in the studies were metal nanoparticles such as selenium, silver, gold, zinc, copper, and iron nanoparticles (*n =* 8, 28.6%), metal oxide nanoparticles such as zinc oxide, titanium dioxide, cerium oxide, zirconium dioxide, and silicon dioxide (*n =* 8, 28.6%), followed by polymeric nanoparticles such as chitosan and chitosan based nanoparticles (*n =* 7, 25.0%). The findings also showed that, in the in vitro studies, the best exposure times were 60 min (*n =* 13, 46.4%), followed by 10 min (*n =* 4, 14.3%) and 120 min (*n =* 3, 10.7%). The results exhibited that the doses used in the in vitro studies were ranging from 0.0005 to 20 mg/kg, whereas in the in vivo studies, the doses ranged from 0.5 to 100 mg/kg.

## 4. Discussion

### 4.1. Preparation Methods of Nanoparticles

According to some factors such as condition of reaction, operation, and approved protocols, several techniques can be used for the nanoparticles synthesis. Currently, there are two main preparation methods, which include (i) bottom-up synthesis or chemo-physical methods such as lipid phase methods (precipitation, sol-gel, hydro-thermal approaches), gas phase methods (flame hydrolysis, spray hydrolysis, and aerosol methods), and biological production by plants, bacteria, fungi, etc., and (ii) top-down synthesis or mechanical-physical methods such as chemical etching, mechanical milling, sputtering, laser ablation, electro-explosion, etc. [44,45,46].

### 4.2. Solid Lipid Nanoparticles (SLNs)

SLNs have been used as a carrier system since the early 1990s. The advantages of these nanoparticles lie with their capability in drug release control, drug targeting, increasing drug chemical stability, acting as a carrier for lipophilic and hydrophilic drugs, having mass production capability, and being completely sterilized. They also increase the bioavailability of praziquantel (PZQ), enhance the pharmacological activity as well as therapeutic efficacy of PZQ and its therapeutic effect and efficacy, and reduce dose and administration frequency [15,47,48].

### 4.3. Albendazole (ABZ)-Loaded SLNs

In a recent study by Aminpour et al., the protoscolicidal effects of albendazole-loaded SLN (ABZ-loaded SLN) were investigated both in vitro and in vivo. In this study, during the seven-day period, the protoscoleces were exposed to the concentrations of 250 and 500 μg/mL of the drug. Then, the number of the remaining cysts was evaluated. On the fifth day of testing, all the protoscoleces in contact with ABZ-loaded SLN disappeared while protoscoleces in contact with the same concentrations of ABZ were destroyed on day 7 of the experiment. On the third day of the experiment, 5 CC of protoscoleces at the concentration of 250 μg/mL ABZ-loaded SLN was injected into the mice and, after three months, the pathogenicity of the protoscoleces in contact with the drug was investigated. It was found that in some of the mice after receiving the above compound, no cysts were formed; the remaining mice had much smaller cysts than the other groups that received ABZ only or did not receive any treatment at all. The results of this study indicated the efficient prophylactic effect of this drug on the treatment of hydatid cysts [15].

### 4.4. Albendazole Sulfoxide-Loaded SLNs

In a study in 2013, Ahmadnia et al. investigated the effects of albendazole sulfoxide-loaded SLN (ALBSO-loaded SLN) on hydatid cysts. The results showed this compound reduced the size of the cysts, but made no significant changes over the duration of this experiment (15 days) and still required more precise experiments, more dosing studies, and longer-term studies [41]. In another study, Soltani et al. (2015) aimed to compare hydatid cyst membrane permeability of ABZ, ABZSO, ABZ-loaded SLN, and ABZSO-loaded SLNs. Their results showed ABZ and ABZSO, due to some unique properties such as good physicochemical characterizations, controlled release, higher permeability, and efficacy by loading into SLNs, were promising for hydatid cyst treatment [17]. In a study published by Rafiei et al. (2019), ultrastructural changes of fertile and infertile cysts exposed to 2 μg/mL of ABZ-loaded SLN and ABZSO-loaded SLNs were investigated. In the histopathologic evaluation of cysts in the control group, which did not receive treatment; in this case, no structural changes were observed, while in the groups treated with the mentioned nanoparticles, the cyst structure was not integrated and only the residues and fragments of protoscoleces were present. The best performance was also observed in the ABZSO-loaded SLNs treated group [16].

### 4.5. Albendazole- and Praziquantile-Loaded SLNs

A 2016 study by Jelowdar et al. on PZQ- and ABZ-loaded SLNs showed that, by giving the drug to mice infected with protoscoleces, the spread of cysts in mice receiving these compounds was much lower than that in the control group receiving only SLNs without the drug. In this group of mice, there was a significant decrease in the size and dry weight of the cysts and the damaged layers of the cysts were highly observed in the mice treated with the above nanoparticles. It should be noted that in comparison to the effect of PZQ- and ABZ-loaded SLNs with free PQZ and ABZ, no significant difference was observed in the number and size of cysts; but, in the destruction rate of cyst layers in the recipient group of PZQ- and ABZ-loaded SLNs, LL loss and serious damage to GL were found. In the group receiving free ABZ and PZQ, only this layer became thin and fragile, and the number of GL cells decreased [40].

### 4.6. Nanolipid Carriers

This combination, created by the change in SLNs, has some advantages. These changes maintain the physical stability of the compound and facilitate the incorporation of more drug into the nanocarier. This compound has been studied in various respects and the results have shown anti-inflammatory, antimicrobial, and efficient drug delivery carriers for anti-cancer drugs [49,50,51].

#### Nanolipid Carriers-Loaded Ivermectin

In 2019, Ahmadpour et al. investigated the effect of nanolipid carriers-loaded ivermectin on the treatment of hydatid cysts. In this study, the concentrations of 200, 500, and 800 μg/mL at 150, 120, and 60 min were able to destroy hydatid cyst protoscoleces, whereas free ivermectin, at concentrations of 800 μg/mL at 150 min, was able to achieve this percentage [19].

### 4.7. Lipid Nanocapsules

LNCs are remarkable structures in the medical field. The benefits of this combination have led to their use not only in therapeutic applications, but also in the fields of drug delivery, cancer diagnosis, and gene and cell therapy. Studies of this compound in the field of cancer have shown promising results, indicating that this compound could reduce tumor mass and increase the cytotoxicity of glioma cells [52].

#### Albendazole-Lipid Nanocapsules

In 2019, Ullio Gamboa et al. investigated the protoscolicidal effects of albendazole-lipid nanocapsules (ABZ LNCs). In this study, various parameters such as LNCs drug payload and encapsulation efficiency, nanoparticle stability, stability of the nanocapsules in simulated gastrointestinal fluids, and animal studies were investigated, all of which had promising results. As seen in the animal studies, 4 out of the 10 infected mice receiving this compound showed no improvement in the cyst development and the size of the cysts decreased significantly compared to the control group. GL was also highly deformed and its cells were reduced [39]. In 2015, Pensela et al. investigated ABZ LNCs in the mice infected with hydatid cysts. In this study, we investigated the plasma and cyst drug exposure after administering ABZ as ABZ-LNCs or ABZ suspension and compared the clinical effects of these two formulations. In the studies following the use of these two formulations, ABZ levels in plasma and cysts were significantly higher when ABZ was used in combination with nanoparticles compared to ABZ suspension [18].

### 4.8. Metal Nanoparticles

Metallic nanoparticles are among the most efficient nanoparticles in various medical fields such as cancer therapy, respiratory disease therapy, neurodegenerative disease therapy, infectious diseases therapy, etc. [43,44]. These nanoparticles have anti-bacterial and biofilm prevention effects by producing ROS, protein adhesion, and membrane destabilization. The anti-cancer effect of these nanoparticles has also been shown to have strong effects on the cancer cells [53,54].

#### 4.8.1. Selenium (Se) NPs

Various studies have been carried out on this nanoparticle, investigating its various aspects. In these studies, we observed a decrease in acute Se toxicity, induction of apoptosis in cancer cells, minimal side-effects on normal cells, and reduction in apoptosis in the diabetic kidney [55,56]. In evaluating the antimicrobial effects of these nanoparticles, these compounds have antimicrobial effects on methicillin-sensitive and methicillin-resistant *Staphylococcus aureus*. Antifungal and antiparasitic effects of these compounds have also been observed. These properties as well as the anti-cancer effects have made these compounds an important part of drug making [57,58].

In 2014, for the first time, the effects of biogenic selenium nanoparticles were evaluated by Mahmoudvand et al. In this study, the concentrations of 500 and 250 μg/mL of this compound for 10 and 20 min had 100% efficacy on the protoscoleces of hydatid cyst, indicating this compound had strong protoscolicidal effects [15]. In 2018, the effect of Se and Ag on the protoscoleces was evaluated by Nematollahi et al. Examining different concentrations of these compounds over 10 to 30 min showed that Se NPs were much stronger than Ag NPs and had approximately 42% yield over 60 min [20].

#### 4.8.2. Silver (Ag) NPs

The antimicrobial and antiparasitic effects of silver nanoparticles have been studied in various studies and promising results have been obtained in cancer-related studies. The antiviral effects of silver nanoparticles have been shown to inhibit replication and binding to the host cell membrane and anti-parasitic studies have demonstrated the inhibition of proliferation and metabolic activity of promastigotes in leishmaniasis. One of the reasons that have led these nanoparticles to attract more attention is their promising effects on developing anticancer drugs [59,60,61,62,63].

In studies on biogenic Ag NPs by Rahimi et al. in 2015, at concentrations of 0.025, 0.05, 0.1, and 0.15 mg/mL, only the concentration of 0.15 mg/mL at 120 min was observed to have 90% protoscolicidal effects. The lowest protoscolicidal activity was also observed at 0.025 mg/mL concentration and 10 min [23]. In addition, Lashkarizadeh et al. reported this nanoparticle in the same year to have poor protoscolicidal effects on the protoscoleces of hydatid cysts, so that at concentrations of 4mg/mL for the period of 60 min, it was able to eliminate 71.6% of protoscoleces, while hypertonic salt solution at a concentration of 20% was able to eliminate 100% of protoscoleces in 10 min [22].

In a recent study by Nassef et al., the therapeutic effects of albendazole-loaded silver nanoparticles along with ABZ and Ag NPs were evaluated. These studies have shown that this compound had the highest pharmacological effect among ABZ and Ag NPs and had the least histopathological effects on the liver. In addition, in the cysts in contact with this nanoparticle, there were marked ultrastructural changes. While measuring size, granuloma size, and weight of cysts, the greatest decrease was in ABZ loaded with silver nanoparticles [38]. In a study by Norouzi et al., the protoscolicidal effects of silver, silica, copper, iron, and zinc nanoparticles were evaluated. In this in vitro work, the highest protoscolicidal effects were related to Ag-NPs at 1 mg/mL concentration after 60 min of exposure time (80% mortality rate), followed by Si-NPs at 1 mg/mL concentration (52.33%), Cu-NPs at 0.5 mg/mL concentration (41%), Fe-NPs at 1mg/mL concentration (28%), and Zn-NPs at concentration of 1mg/mL after 60 min (15.67%) [24].

#### 4.8.3. Gold (Au) NPs

Gold nanoparticles are among the most important compounds in the medical field. There have been numerous studies on the treatment of important parasitic diseases such as leishmaniasis and malaria that have shown successful results. In addition, applying these nanoparticles for identifying different parasites has also been studied and confirmed. Gold nanoparticles have been shown to have a highly stable and adaptable structure for drug delivery, which has increased the interest in these nanoparticles [64,65].

In the recent study by Napooni on the protoscolicidal effects of this nanoparticle, we observed that the highest lethal effect was at 60-min exposure to protoscoleces with 4000 μg/mL gold nanoparticles. Furthermore, the cytotoxicity of this compound was found to be low at all concentrations. Ultrastructure changes in the tegument, shape of the sucker, size, and DNA fragmentation were also observed in protoscoleces [26]. Another study in 2016 on these nanoparticles by Barabadi et al. showed that the in vitro use of 300 μg/mL of these nanoparticles compared to other groups (groups receiving 5% normal saline and concentrations of 50, 100, and 200 μg/mL gold nanoparticles) had the highest removal rate of protoscoleces in 2 h [25]. In a recent study by Çolak et al., the protoscolicidal activity of AuNPs at concentrations of 0.4 and 0.8 mL and three laser powers including 30, 50, and 150 mW were evaluated for 30, 60, and 120 min. They found that 89.3% of the protoscoleces was killed after treatment with AuNPs under high dose (150 mW) laser power for 120 min, indicating that increasing the dose of AuNPs or laser power or used time increased the mortality rate of protoscoleces [43].

Malekifard et al. (2017) also investigated the protoscolicidal effects of these nanoparticles. Protoscolicidal effects of gold nanoparticles were studied in vitro at concentrations of 250, 500, and 1000 μg/mL for 5 to 60 min in contact with hydatid cyst fluid. Gold nanoparticles at all concentrations used had significant protoscolicidal effects compared to the control group, so that all the protoscoleces in contact with the concentration of 1000 μg/mL were eliminated within 1 h [27].

### 4.9. Non-Metal NPs

#### ABZ-Loaded Nanoparticles

In a study in 2008, Truong Cong et al. evaluated the diffusion ability of ABZ nanoparticles and increased drug concentration in hydatid cysts. A good correlation was observed between the permeation coefficient and partition coefficient. The drug release from the nanoparticles through the hydatid membrane was improved compared to the soluble ABZ and showed sufficient entrapment efficiency to increase the apparent solubility of ABZ [42].

### 4.10. Metal Oxide NPs

#### 4.10.1. Zinc Oxide Nanoparticles

ZnO nanoparticles are non-toxic and compatible with body skin. Properties such as skin adaptation, non-toxicity, antimicrobial, antiparasitic, and antifungal activity have made these nanoparticles a widely-used and important compound in manufacturing medicine. These nanoparticles, without toxic effects on healthy cells, induce death in cancer cells; studies have shown that these nanoparticles can be used for gene delivery [66,67].

Norouzi et al. recently investigated the protoscolicidal effects of zinc oxide nanoparticles for the first time. At 50 and 100 mg/mL concentrations, the highest protoscolicidal effects of this compound were observed in 10 min of zinc oxide and protoscolece nanoparticles exposure, which was 19.6% (very weak) and with no increase in performance over time; it was a low percentage, indicating this compound was not suitable for use in hydatid cyst surgery [28]. In another study on this nanoparticle in 2015 by Razi Jalali et al., the protoscolicidal effects of zinc oxide and ABZ, *Echinacea purpurea*, and *Sambucus ebulus* nanoparticles were investigated, which revealed a decrease in size, volume, and number of cysts in all the compounds [37].

#### 4.10.2. Titanium Dioxide (TiO2) Nanoparticles

TiO2 nanoparticles are considered as one of the most widely used nanoparticles in various industries such as cosmetics, food, and pharmaceuticals, since they have some physicochemical properties such as non-toxicity, cost-effectiveness, anticorrosive, high stability, photocatalytic properties, etc. These compounds kill cells by inactivating germ cell and DNA enzymes as well as by removing fluids from bacterial cells. Other uses of these compounds include treating and diagnosing cancer [68,69,70]. Navvabi et al. (2019) recently investigated the in vitro and in viovo protoscolicidal effects of *Echinometra mathaei* (sea urchin gonad) extract alone or combined with TiO2 NPs. Their results showed that sea urchin gonad extract at the concentration 15 μg/mL, especially in combination with TiO2, killed 84% of the protoscoleces after 60 min exposure in vitro. On the other hand, oral administration of infected mice with the combination of the gonad extract + TiO2 for three months demonstrated higher efficacy by reduction in number, size and volume of the hydatid cysts in comparison to the control group [29].

#### 4.10.3. Cerium Dioxide Nanoparticles

Cerium (Ce) is one of the rare-earth elements that, because of its special structure, has expended its use. Other features include its cost-effectiveness. Many studies have investigated the benefits of this nanoparticle, such as antimicrobial, anti-cancer, treatment, and antioxidant activities [71,72]. In a study by Aryamand et al. (2019), the in vitro and in vivo protoscolicidal effects of *Holothuria leucospilota* extract alone, CeO2 nanoparticles alone, and extract combined with CeO2 NPs were investigated for 10 to 60 min. In vitro results showed that the most protoscolicidal effects were reported for extract (70% at 20 mg/mL for 60 min), followed by the combination of this extract and Ce nanoparticles (63% in 15 mg/mL concentration for 1 h). Furthermore, in vivo assay demonstrated that all three compounds significantly reduced the number and size of the hydatid cysts compared to the control group that received no treatment [30].

#### 4.10.4. Zirconium Dioxide Nanoparticles

Zirconium (Zr) is a chemical element which has some applications, especially in medicine and dentistry [73]. Zirconium dioxide (ZrO2), also called zirconia, has some unique properties such as high compatibility, low toxicity, low cost, and high strength and is broadly used in various biomedical fields including antimicrobial ones [74,75,76,77]. In a study conducted by Ibrahim (2020), ZrO2 at concentrations of 1000, 2000, and 4000 µg/mL significantly killed 49.6, 52.7, and 53.1% of the hydatid protoscoleces after 60 min [31].

### 4.11. Nanopolymeric Particles

Various properties of chitosan nanoparticles (Ch NPs) including non-toxicity, water solubility, stability, simple preparation, environmental compliance, and antimicrobial activity have made them remarkable and effective compounds in the field of medicine. In the field of vaccine development, several studies have been performed to evaluate the usefulness of these nanoparticles, showing their beneficial effects [78,79,80].

#### 4.11.1. Chitosan-Curcumin Nanoparticles

In another study by Napooni et al. (2019), the in vitro protoscolicidal effects of chitosan–curcumin nanoparticle (Ch-Cu NPs) at different concentrations of 0.25, 0.05, 1, 2, and 4 mg/mL were evaluated for 5, 10, 20, 30, and 60 min. The results showed that the highest mortality rate (68%) of protoscoleces was observed after exposure to Ch-Cu-NPs at a concentration of 4000 µg/mL for 60 min, whereas by scanning electron microscopy, the length and width of protoscoleces were significantly reduced compared to the control group [32].

#### 4.11.2. Chitosan-Praziquantel and -Albendazole Nanoparticles

Torabi et al. (2018) examined the protoscolicidal, prophylactic, and therapeutic effects of ChPZQ and ChABZ. In evaluating the in vitro protoscolicidal effects of these compounds, microcysts were exposed (for 16 days) at concentrations of 1, 5, and 10 μg/mL of chitosan-praziquantel (ChPZQ) and -albendazole (ChABZ); then, it was observed that the best effect during this time was related to using these two compounds together (at concentrations 5 and 10) when no microcysts were observed for 10 days post-incubation. Compared to ChPZQ and ChABZ, ChPZQ performed better at all the three concentrations than ChABZ (no significant difference). In order to evaluate the therapeutic and prophylactic effects of these compounds, the number and weight of cysts in contact with the above compounds were evaluated. For evaluating the prophylactic effect of these compounds, a significant decrease in the number and weight of cysts in the group receiving the two compounds was observed compared to the control group receiving no medication. However, in the evaluation of therapeutic effect, there was a significant difference in the number of cysts in the group receiving ChABZ and ChPZQ nanoparticles together compared to the control group; but no significant decrease was observed in the weight of the cysts compared to the control group. In the mice receiving both combinations, GL and LL were separated [81]. Furthermore, in another study by Torabi et al., ChPZQ showed more stability than ChABZ, which could be due to its better performance [66].

In a recent study conducted by Darvishi et al. (2020), the effects of ABZ-sulfoxide (SO)-loaded chitosan (CS)-PGLA NPs synthesized by nanoprecipitation orally administered at a dose of 10 mg/kg/day for 45 days showed significant therapeutic effect in the weight and volume of cysts in comparison to that in the control group, indicating that ABZ-SO-loaded CS-PGLA NPs could improve the therapeutic effects of ABZ-SO in the CE treatment in mice [36].

#### 4.11.3. Albendazole Sulfoxide-Loaded PLGA-PEG NPs

In 2016, Naseri et al. investigated the protoscolicidal effects and apoptotic activity of albendazole sulfoxide-loaded PLGA-PEG (ABZs-loaded PLGA-PEG). In this study, concentrations of 50, 100, 150, and 200 μg/mL of these compounds were exposed to a specific concentration of hydatid cyst fluid for 5 to 60 min. To evaluate the protoscolicidal effects, it was observed that at concentrations of 150 and 200 μg/mL, the nanodrug (at all times of the experiment) had 100% protoscolicidal effects, while at a concentration of 200 μg/mL, albendazole at 30 min had a 100% effect. Protoscoleces treated with ABZs-loaded PLGA-PEG showed surface shrinkage, disoriented appearance, and a disrupted characteristic due to programmed cell death. Both compounds had apoptotic intensity, but no significant difference was observed in the activity of both compounds [34].

#### 4.11.4. Flubendazole-Loaded mPEG-PCL NPs

In 2018, studies on the protoscolicidal effects of flubendazole-loaded mPEG-PCL NPs were conducted by Farhadi et al. The in vitro study showed that, at exposure time of 27 days, 10 μg/mL of these nanoparticles was able to kill all the protoscoleces on the 15th day. In vivo studies also showed that the number of cysts was significantly lower than the control group, but the difference in the number of cysts in the free flobendazole recipient group and the group that received nanoparticle was not significant. The weight of the cysts in the nanoparticle receiving group was much lower than the other groups; the cysts underwent many changes and there were marked ultrastructural changes in the germinal layer [35].

## 5. Conclusions

The results of this review study show the high efficacy of a wide range of organic and inorganic NPs against CE, indicating that nanoparticles could be considered as an alternative and complementary resource for CE treatment. The results demonstrated that the most widely used nanoparticles for hydatid cyst treatment are metal nanoparticles, metal oxide nanoparticles, followed by polymeric nanoparticles. We found that the most compatible drugs with nanoparticles were albendazole, followed by praziquantel and flubendazole, indicating a deeper understanding about the synergistic effects of nanoparticles and the present anti-parasitic drugs to treat hydatid cysts. The important point about using these nanoparticles is their toxicity; therefore, cytotoxicity as well as acute and chronic toxicities of these nanoparticles should be considered in particular. As a limitation, in the present study, although most studies have been performed in vitro, more studies are needed to confirm the effect of these nanoparticles as well as their exact mechanisms in hydatid cyst treatment, especially in animal models and clinical settings.

## Figures and Tables

**Table 1:** A list of in vitro studies on effects of nanoparticles against hydtid cysts.

Nanoparticles	Drug		Outcome		Ref
			Concentration of Drug (µg/mL)	Best Exposure Time	
Solid lipid nanoparticles (SLNs)	Albendazole Loaded SLN		250	Fifth day	[15]
			500	Fifth day	
			2	72 h	[16]
	Albendazole Sulfoxide Loaded SLN		2 2.5	72 h	[17]
			2	72 h	[16]
Lipid nanocapsules (LNCs)	Albendazole -LNCs		0.5	-	[18]
			1	-	
			1.5	7 day	
Nano lipid carriers (NLCs)	NLCs Loaded Ivermectin		50	-	[19]
			100	-	
			200	150 min	
			400	120 min	
			800	60 min	
Metal NPs	Selenium NPs		50	-	[20]
			125	-	
			250	20 min	
			500	10 min	
			50	-	[21]
			125	-	
			250	-	
			500	60 min	
	Silver NPs		500	-	[22]
			1000	-	
			2000	-	
			4000	60 min	
			25	-	[23]
			50	-	
			100	-	
			150	120 min	
			50	-	[21]
			125	-	
			250	60 min	
			500	-	
			250	-	[24]
			500	-	
			1000	60 min	
	Gold NPs		50	-	[25]
			100	-	
			200	-	
			300	120 min	
			250	-	[26]
			500	-	
			1000	-	
			2000	-	
			4000	60 min	
			250	-	[27]
			500	-	
			1000	60 min	
	Zinc NPs		250	10 min	[24]
			500	-	
			1000	-	
	Copper NPs		250	-	[24]
			500	60 min	
			1000	-	
	Iron NPs		250	-	[24]
			500	10 min	
			1000	-	
Metal oxide NPs	Zinc Oxide NPs		50,000	10 min	[28]
			10,000	-	
	Sea Urchin Gonad Extraction Combined with Tio2 NPs		1	-	[29]
			5	-	
			15	60 min	
	CeO2 NPs		1000	-	[30]
			5000	-	
			10,000	-	
			15,000	-	
			20,000	60 min	
	ZrO2		250	-	[31]
			500	-	
			1000	60 min	
			2000	60 min	
			4000	60 min	
Nonmetals oxide NPs	SiO2 NPs		250	-	[24]
			500	-	
			1000	60 min	
Nanopolymeric particles	Chitosan NPs	Curcumin	500	-	[32]
			1000	-	
			2000	-	
			4000	60 min	
		Praziquantel	1	-	[33]
			5	Tenth days	
			10	Tenth days	
		Albendazole	1	-	[33]
			5	Tenth days	
			10	Tenth days	
	Albendazole Sulfoxide Loaded PLGA-PEG NPs		50	-	[34]
			100	-	
			150	All The time	
			200	All The time	
	Flubendazole-Loaded mPEG-PCL NPs		1	-	[35]
			5	-	
			10	Fifteenth days	

**Table 2:** A list of in vivo studies on effects of nanoparticles against hydtid cysts.

Group	Drug		Animal	Outcome			Ref.
				Drug Dose	Duration of Medication	Investigation Timeframe	
Nanopolymeric particles	Flubendazole-loaded mPEG-PCL NPs		BALB/c mice	5 mg/kg	1 month	8 months	[35]
	Chitosan NPs	Praziquantel	Male DBA/2 mice	25mg/kg	21 days	8 months	[33]
		Albendazole	Male DBA/2 Mice	25 mg/kg	21 days	8 months	[33]
		Albendazole sulfoxide (ABZ-SO)–loaded chitosan-PLGA nanoparticles	BALB/c mice	10 mg/kg	45 days	10 months	[36]
Metal oxide NPs	CeO2 NPs		Male BALB/c mice	50 mg/kg	1 month	2 months	[30]
	Sea Urchin Gonad Extraction Combined with Tio2 NPs		BALB/c mice	-	3 month	3 months	[29]
	Zinc Oxide NPs						[37]
Metal NPs	Albendazole-Loaded Silver NPs		Female albino mice	100 mg/kg	2 month	2 months	[38]
	Silver NPs		Female albino mice	25 mg/kg	2 month	2 months	[38]
Lipid Nanocapsules (LNCs)	Albendazole -LNCs		Female CF-1 mice	5 mg/kg	1 month	6 months	[39]
			Female CF-1 mice	5 mg/kg	1 month	8 months	[18]
Solid lipid nanoparticles (SLNs)	Praziquantel Loaded SLN		Female BALB/c mice	-	3 month	5 months	[40]
	Albendazole Loaded SLN		Female BALB/c mice	-	3 month	5 months	[40]
	Albendazole Sulfoxide Loaded SLN		Male BALB/c mice	0.5 mg/kg 2 mg/kg	15 day	8 months	[41]
	Albendazole Loaded SLN		BALB/c mice	-	-	3 months	[15]

**Table 3:** A list of ex vivo studies on effects of nanoparticles against hydtid cysts.

Group	Drug	Outcome		Ref
		Concentration of Drug (mL)	Best Exposure Time	
Nonmetal Nanoparticles	Albendazole Loaded NPs	0.5	-	[42]
Metal Nanoparticles	Gold NPs	0.4	-	[43]
		0.8	120 min	

## Data Availability

All data generated or analyzed during this study are included in this published article.

## References

[1] Mcmanus D.P., Zhang W., Li J., Bartley P.B. (2003). Echinococcosis. Lancet.

[2] Eckert J., Deplazes P. (2004). Biological, Epidemiological, and Clinical Aspects of Echinococcosis, a Zoonosis of Increasing Concern. Clin. Microbiol. Rev..

[3] Brunetti E., Kern P., Vuitton D.A. (2010). Expert consensus for the diagnosis and treatment of cystic and alveolar echinococcosis in humans. Acta Trop..

[4] Junghanss T., Da Silva A.M., Horton J., Chiodini P.L., Brunetti E. (2008). Clinical management of cystic echinococcosis: State of the art, problems, and perspectives. Am. J. Trop. Med. Hyg..

[5] Eckert J. (1996). Guidelines for treatment of cystic and alveolar echinococcosis in humans. Bull. World Health Organ..

[6] Dehkordi A.B., Sanei B., Yousefi M., Sharafi S.M., Safarnezhad F., Jafari R., Darani H.Y. (2018). Albendazole and treatment of hydatid cyst, review of literature. Infect. Disord. Drug Targets.

[7] Sahin M., Eryilmaz R., Bulbuloglu E. (2004). The Effect of Scolicidal Agents on Liver and Biliary Tree (Experimental Study). J. Investig. Surg..

[8] Mishra S. (2016). Nanotechnology in medicine. Indian Heart J..

[9] Zhu X., Radovic-Moreno A.F., Wu J., Langer R., Shi J. (2014). Nanomedicine in the management of microbial infection—Overview and perspectives. Nano Today.

[10] Sengul A.B., Asmatulu E. (2020). Toxicity of metal and metal oxide nanoparticles: A review. Environ. Chem. Lett..

[11] Salata O.V. (2004). Applications of nanoparticles in biology and medicine. J. Nanobiotechnol..

[12] Rajput N. (2015). Methods of preparation of nanoparticles—A review. Int. J. Adv. Eng. Technol..

[13] Shnawa B.H. (2018). Advances in the Use of Nanoparticles as Anti-Cystic Echinococcosis Agents: A Review Article. J. Pharm. Res. Int..

[14] Moher D., Liberati A., Tetzlaff J., Altman D.G., Prisma Group (2009). Preferred reporting items for systematic reviews and meta-analyses: The PRISMA statement. PLoS Med..

[15] Aminpour S., Rafiei A., Jelowdar A., Kouchak M. (2019). Evaluation of the Protoscolicidal Effects of Albendazole and Albendazole Loaded Solid Lipid Nanoparticles. Iran. J. Parasitol..

[16] Rafiei A., Soltani S., Ramezani Z., Abbaspour M.R., Jelowdar A., Kahvaz M.S. (2019). Ultrastructural changes on fertile and infertile hydatid cysts induced by conventional and solid lipid nanoparticles of albendazole and albendazole sulfoxide. Comp. Clin Path..

[17] Soltani S., Rafiei A., Ramezani Z., Abbaspour M.R., Jelowdar A., Kahvaz M.S. (2017). Evaluation of the hydatid cyst membrane permeability of albendazole and albendazole sulfoxide-loaded solid lipid nanoparticles. Jundishapur J. Nat. Pharm. Prod..

[18] Pensel P.E., Gamboa G.V.U., Fabbri J., Ceballos L., Bruni S.S., Alvarez L.I., A Allemandi D., Benoît J., Palma S.D., Elissondo M.C. (2015). Cystic echinococcosis therapy: Albendazole-loaded lipid nanocapsules enhance the oral bioavailability and efficacy in experimentally infected mice. Acta Trop..

[19] Ahmadpour E., Godrati-Azar Z., Spotin A., Norouzi R., Hamishehkar H., Nami S., Heydarian P., Rajabi S., Mohammadi M., Perez-Cordon G. (2019). Nanostructured lipid carriers of ivermectin as a novel drug delivery system in hydatidosis. Parasites Vectors.

[20] Mahmoudvand H., Harandi M.F., Shakibaie M., Aflatoonian M.R., Zia Ali N., Makki M.S., Jahanbakhsh S. (2014). Scolicidal effects of biogenic selenium nanoparticles against protoscoleces of hydatid cysts. Int. J. Surg..

[21] Nematollahi A., Shahbazi P., Rafat A., Ghanbarlu M. (2018). Comparative survey on scolicidal effects of selenium and silver nanoparticles on protoscoleces of hydatid cyst. Open Vet. J..

[22] Lashkarizadeh M.R., Asgaripour K., Dezaki E.S., Harandi M.F. (2015). Comparison of Scolicidal Effects of Amphotricin B, Silver Nanoparticles, and Foeniculum vulgare Mill on Hydatid Cysts Protoscoleces. Iran. J. Parasitol..

[23] Rahimi M.T., Ahmadpour E., Esboei B.R., Spotin A., Koshki M.H.K., Alizadeh A., Honary S., Barabadi H., Mohammadi M.A. (2015). Scolicidal activity of biosynthesized silver nanoparticles against Echinococcus granulosus protoscoleces. Int. J. Surg..

[24] Norouzi R., Ataei A., Hejazy M., Noreddin A., Ezzat M., Zowalaty E. (2020). Scolicidal Effects of Nanoparticles Against Hydatid Cyst Protoscoleces in vitro. Int. J. Nanomed..

[25] Barabadi H., Honary S., Ali Mohammadi M., Ahmadpour E., Rahimi M.T., Alizadeh A., Naghibi F., Saravanan M. (2017). Green chemical synthesis of gold nanoparticles by using Penicillium aculeatum and their scolicidal activity against hydatid cyst protoscoleces of Echinococcus granulosus. Environ. Sci. Pollut. Res..

[26] Napooni S., Arbabi M., Delavari M., Hooshyar H., Rasti S. (2019). Lethal effects of gold nanoparticles on protoscoleces of hydatid cyst: In vitro study. Comp. Clin. Path..

[27] Malekifard F. (2017). Solicidal effect of the gold nanoparticle on protoscoleces of hydratid cyst in vitro. J. URMIA Univ. Med. Sci..

[28] Norouzi R., Hejazy M., Ataei A. (2019). Scolicidal effect of zinc oxide nanoparticles against hydatid cyst protoscoleces in vitro. Int. J. Nanomed..

[29] Navvabi A., Homaei A., Khademvatan S., Ansari M.H.K., Keshavarz M. (2019). Combination of TiO_2_ nanoparticles and Echinometra mathaeis gonad extracts: In vitro and in vivo scolicidal activity against hydatid cysts. Biocatal. Agric. Biotechnol..

[30] Aryamand S., Khademvatan S., Tappeh K.H., Heshmatian B., Jelodar A. (2019). In Vitro and in Vivo Scolicidal Activities of Holothuria leucospilota Extract and CeO_2_ Nanoparticles against Hydatid Cyst. Iran. J. Parasitol..

[31] Ibrahim A.A.J. (2020). Scolicidal Activity of Zirconium Oxide (ZrO_2_) nanoparticles Against Protoscolices of Hydatid Cysts. Indian J. Forensic Med. Toxicol..

[32] Napooni S., Delavari M., Arbabi M., Barkheh H., Rasti S., Hooshyar H., Mashkani S.M.H. (2019). Scolicidal Effects of Chitosan–Curcumin Nanoparticles on the Hydatid Cyst Protoscoleces. Acta Parasitol..

[33] Torabi N., Dobakhti F., Faghihzadeh S., Haniloo A. (2018). In vitro and in vivo effects of chitosan-praziquantel and chitosan-albendazole nanoparticles on Echinococcus granulosus Metacestodes. Parasitol. Res..

[34] Naseri M., Akbarzadeh A., Spotin A., Akbari N.A.R., Mahami-Oskouei M., Ahmadpour E. (2016). Scolicidal and apoptotic activities of albendazole sulfoxide and albendazole sulfoxide-loaded PLGA-PEG as a novel nanopolymeric particle against Echinococcus granulosus protoscoleces. Parasitol. Res..

[35] Farhadi M., Haniloo A., Rostamizadeh K., Faghihzadeh S. (2018). Efficiency of flubendazole-loaded mPEG-PCL nanoparticles: A promising formulation against the protoscoleces and cysts of Echinococcus granulosus. Acta Trop..

[36] Darvishi M.M., Moazeni M., Alizadeh M., Abedi M., Tamaddon A.-M. (2020). Evaluation of the efficacy of albendazole sulfoxide (ABZ-SO)–loaded chitosan-PLGA nanoparticles in the treatment of cystic echinococcosis in laboratory mice. Parasitol. Res..

[37] Razi J.M., Alborzi A., Najafzade Varzi H., Ghorbanpour M., Derakhshan L. (2015). Survey on effects of albendazole, echinacea purpurea, sambucus ebulus and zinc oxide nanoparticles on unilocular hydatid cyst in mice. Sci. Iran. Vet. J..

[38] Nassef N.E., Saad A.-G.E., Harba N.M., Beshay E.V.N., Gouda M.A., Shendi S.S., Mohamed A.S.E.-D. (2019). Evaluation of the therapeutic efficacy of albendazole-loaded silver nanoparticles against Echinococcus granulosus infection in experimental mice. J. Parasit. Dis..

[39] Gamboa G.V.U., Pensel P.E., Elissondo M.C., Bruni S.F.S., Benoît J., Palma S.D., Allemandi A. (2019). Albendazole-lipid nanocapsules: Optimization, characterization and chemoprophylactic efficacy in mice infected with Echinococcus granulosus. Exp. Parasitol..

[40] Jelowdar A., Rafiei A., Abbaspour M., Rashidi I., Rahdar M. (2017). Efficacy of combined albendazol and praziquntel and their loaded solid lipid nanoparticles components in chemoprophylaxis of experimental hydatidosis. Asian Pac. J. Trop. Biomed..

[41] Ahmadnia S., Moazeni M., Mohammadi-Samani S., Oryan A. (2013). In vivo evaluation of the efficacy of albendazole sulfoxide and albendazole sulfoxide loaded solid lipid nanoparticles against hydatid cyst. Exp. Parasitol..

[42] Cong T.T., Faivre V., Nguyen T.T., Heras H., Pirot F., Walchshofer N., Sarciron M.-E., Falson F. (2008). Study on the hydatid cyst membrane: Permeation of model molecules and interactions with drug-loaded nanoparticles. Int. J. Pharm..

[43] Çolak B., Aksoy F., Yavuz S., Demircili M.E. (2019). Investigating the effect of gold nanoparticles on hydatid cyst protoscoleces under low-power green laser irradiation. Turk. J. Surg..

[44] Okuyama K., Lenggoro I.W. (2003). Preparation of nanoparticles via spray route. Chem. Eng. Sci..

[45] Jahn A., Reiner J.E., Vreeland W.N., DeVoe D.L., Locascio L.E., Gaitan M. (2008). Preparation of nanoparticles by continuous-flow microfluidics. J. Nanoparticle. Res..

[46] Khan I., Saeed K., Khan I. (2019). Nanoparticles: Properties, applications and toxicities. Arab. J. Chem..

[47] Li Y., Yehui G., Hao L., Yu Z., Jinsong Y., Yanyan C. (2009). Enhancement the oral bioavailability of praziquantel by incorporation into solid lipid nanoparticles. Pharmazie.

[48] Xie S., Pan B., Shi B., Zhang Z., Zhang X., Wang M., Zhou W. (2011). Solid lipid nanoparticle suspension enhanced the therapeutic efficacy of praziquantel against tapeworm. Int. J. Nanomed..

[49] Purohit D.K., Nandgude T.D., Poddar S.S. (2016). Nano-lipid carriers for topical application: Current scenario. Asian J. Pharm..

[50] Beloqui A., Solinís M.Á., Rodríguez-Gascón A., Almeida A.J., Préat V. (2016). Nanostructured lipid carriers: Promising drug delivery systems for future clinics. Nanomed. Nanotechnol. Biol. Med..

[51] Cortesi R., Valacchi G., Muresan X.M., Drechsler M., Contado C., Esposito E., Grandini A., Guerrini A., Forlani G., Sacchetti G. (2017). Nanostructured lipid carriers (NLC) for the delivery of natural molecules with antimicrobial activity: Production, characterisation and in vitro studies. J. Microencapsul..

[52] Huynh N., Passirani C., Saulnier P., Benoit J.P. (2009). Lipid nanocapsules: A new platform for nanomedicine. Int. J. Pharm..

[53] Conde J., Doria G., Baptista P. (2012). Noble Metal Nanoparticles Applications in Cancer. J. Drug Deliv..

[54] Gold K., Slay B., Knackstedt M., Gaharwar A.K. (2018). Antimicrobial Activity of Metal and Metal-Oxide Based Nanoparticles. Adv. Ther..

[55] Kumar G.S., Kulkarni A., Khurana A., Kaur J., Tikoo K. (2014). Selenium nanoparticles involve HSP-70 and SIRT1 in preventing the progression of type 1 diabetic nephropathy. Chem. Biol. Interact..

[56] Khurana A., Tekula S., Saifi M.A., Venkatesh P., Godugu C. (2019). Therapeutic applications of selenium nanoparticles. Biomed. Pharmacother..

[57] Wadhwani S.A., Shedbalkar U.U., Singh R., Chopade B.A. (2016). Biogenic selenium nanoparticles: Current status and future prospects. Appl. Microbiol. Biotechnol..

[58] Huang T., Holden J.A., Heath D.E., O’Brien-Simpson N.M., O’Connor A.J. (2019). Engineering highly effective antimicrobial selenium nanoparticles through control of particle size. Nanoscale.

[59] Wei L., Lu J., Xu H., Patel A., Chen Z.-S., Chen G. (2015). Silver nanoparticles: Synthesis, properties, and therapeutic applications. Drug Discov. Today.

[60] Gaafar M.R., Mady R., Diab R., Shalaby T. (2014). Chitosan and silver nanoparticles: Promising anti-toxoplasma agents. Exp. Parasitol..

[61] Allahverdiyev A., Abamor E.Ş., Bagirova M., Ustundag C.B., Kaya C., Rafailovich M. (2011). Antileishmanial effect of silver nanoparticles and their enhanced antiparasitic activity under ultraviolet light. Int. J. Nanomed..

[62] Galdiero S., Falanga A., Vitiello M., Cantisani M., Marra V., Galdiero M. (2011). Silver Nanoparticles as Potential Antiviral Agents. Molecules.

[63] Dos Santos C.A., Rai M., Ingle A.P., Gupta I., Galdiero S., Galdiero M., Gade A., Rai M. (2014). Silver Nanoparticles: Therapeutical Uses, Toxicity, and Safety Issues. J. Pharm. Sci..

[64] Thambiraj S., Hema S., Shankaran D.R. (2018). Functionalized gold nanoparticles for drug delivery applications. Mater. Today Proc..

[65] Benelli G. (2018). Gold nanoparticles—Against parasites and insect vectors. Acta Trop..

[66] Webster T.J.T., Taylor E. (2011). Reducing infections through nanotechnology and nanoparticles. Int. J. Nanomed..

[67] Mirzaei H., Darroudi M. (2017). Zinc oxide nanoparticles: Biological synthesis and biomedical applications. Ceram. Int..

[68] Çeşmeli S., Avci C.B. (2019). Application of titanium dioxide (TiO_2_) nanoparticles in cancer therapies. J. Drug Target..

[69] Alhadrami H.A., Baqasi A., Iqbal J., Shoudri R.A., Ashshi A.M., Azhar E.I., Al-Hazmi F., Al-Ghamdi A., Wageh S. (2017). Antibacterial Applications of Anatase TiO_2_ Nanoparticle. Am. J. Nanomater..

[70] Peiris M.M.K., Guansekera T.D.C.P., Jayaweera P.M., Fernando S.S.N. (2018). TiO_2_ nanoparticles from baker’s yeast: A potent antimicrobial. J. Microbiol. Biotechnol..

[71] Dhall A., Self W.T. (2018). Cerium Oxide Nanoparticles: A Brief Review of Their Synthesis Methods and Biomedical Applications. Antioxidants.

[72] Nithya P., Sundrarajan M. (2020). Ionic liquid functionalized biogenic synthesis of Ag[sbnd]Au bimetal doped CeO_2_ nanoparticles from Justicia adhatoda for pharmaceutical applications: Antibacterial and anti-cancer activities. J. Photochem. Photobiol. B Biol..

[73] Chen Y.-W., Moussi J., Drury J.L., Wataha J.C. (2016). Zirconia in biomedical applications. Expert Rev. Med. Devices.

[74] Larsson C. (2011). Zirconium dioxide based dental restorations. Studies on clinical performance and fracture behaviour. Swed. Dent. J. Suppl..

[75] Patil N.A., Kandasubramanian B. (2020). Biological and mechanical enhancement of zirconium dioxide for medical applications. Ceram. Int..

[76] Fathima J.B., Pugazhendhi A., Venis R. (2017). Synthesis and characterization of ZrO_2_ nanoparticles-antimicrobial activity and their prospective role in dental care. Microb. Pathog..

[77] Gowri S., Gandhi R.R., Sundrarajan M. (2014). Structural, Optical, Antibacterial and Antifungal Properties of Zirconia Nanoparticles by Biobased Protocol. J. Mater. Sci. Technol..

[78] Tiyabooncjai W., Tiyaboonchai W. (2003). Chitosan nanoparticles: A promising system for drug delivery. Naresuan Univ. J..

[79] Illum L., Jabbal-Gill I., Hinchcliffe M., Fisher A., Davis S. (2001). Chitosan as a novel nasal delivery system for vaccines. Adv. Drug Deliv. Rev..

[80] Divya K., Jisha M. (2018). Chitosan nanoparticles preparation and applications. Environ. Chem. Lett..

[81] Torabi N., Dobakhti F., Haniloo A. (2018). Albendazole and Praziquantel Chitosan Nanoparticles: Preparation, Characterization, and In Vitro Release Study. Iran. J. Sci. Technol. Trans. A Sci..

